# Hydrogen Peroxide Is Involved in Salicylic Acid-Elicited Rosmarinic Acid Production in *Salvia miltiorrhiza* Cell Cultures

**DOI:** 10.1155/2014/843764

**Published:** 2014-05-29

**Authors:** Wenfang Hao, Hongbo Guo, Jingyi Zhang, Gege Hu, Yaqin Yao, Juane Dong

**Affiliations:** State Key Laboratory of Crop Stress Biology for Arid Areas, College of Life Sciences, Northwest A&F University, Yangling 712100, China

## Abstract

Salicylic acid (SA) is an elicitor to induce the biosynthesis of secondary metabolites in plant cells. Hydrogen peroxide (H_2_O_2_) plays an important role as a key signaling molecule in response to various stimuli and is involved in the accumulation of secondary metabolites. However, the relationship between them is unclear and their synergetic functions on accumulation of secondary metabolites are unknown. In this paper, the roles of SA and H_2_O_2_ in rosmarinic acid (RA) production in *Salvia miltiorrhiza* cell cultures were investigated. The results showed that SA significantly enhanced H_2_O_2_ production, phenylalanine ammonia-lyase (PAL) activity, and RA accumulation. Exogenous H_2_O_2_ could also promote PAL activity and enhance RA production. If H_2_O_2_ production was inhibited by NADPH oxidase inhibitor (IMD) or scavenged by quencher (DMTU), RA accumulation would be blocked. These results indicated that H_2_O_2_ is secondary messenger for signal transduction, which can be induced by SA, significantly and promotes RA accumulation.

## 1. Introduction

Salicylic acid (SA) is often used to regulate plant growth and development, seed germination, and fruit formation and to enhance the capability of the plants to respond to abiotic and biotic stresses [1, 2]. Exogenous application of SA can promote the thermo tolerance of mustard seedlings [3], chilling tolerance of cucumber [4], salt stress of* Arabidopsis* seedlings [5], and toxicity tolerance of cadmium of barley seedlings [6]. In recent years, SA has been used as an elicitor to induce the biosynthesis of secondary metabolites in plants. Exogenous application of SA induces the biosynthesis of coumarins in* Matricaria chamomilla* [7], taxane in* Taxus chinensis* [8], saponins in ginseng [9], phenolic acids in* Salvia miltiorrhiza* [10], artemisinin in* Artemisia annua* [11], and sinapyl alcohol in ulmus cells [12].

The biosynthesis of secondary metabolites in plant cells is regulated by specific signal transduction pathways. Under the stimulation of elicitors, some biochemical events related to signal transduction can be activated, such as ion transmembrane transport, active oxide species (AOS) burst, and protein phosphorylation and dephosphorylation [13, 14]. AOS are signal molecules that widely exist in plant cells [13–15]. Hydrogen peroxide (H_2_O_2_), one of the AOS, has been considered as the most significant signal molecules [14, 16]. Generally, when plants are under stress, H_2_O_2 _is produced rapidly to activate systemic acquired resistance [13, 17, 18] and acts as a signal molecule mediating the biosynthesis of secondary metabolites in plants. For example, H_2_O_2_ is the key signal molecule for oligosaccharides to induce the biosynthesis of taxol in* T. chinensis* cells [19], for the cell wall of* Aspergillus niger *to elicit* Catharanthus roseus* cells to synthesize catharanthine [20], for ABA to induce rice seedling leaves to synthesize anthocyanidins [21], and for the cell cultures of* Rubia tinctorum* to produce anthraquinone [22], and so forth.

Rosmarinic acid (RA) is one of effective compounds of Danshen, the root and rhizome of* Salvia miltiorrhiza* Bunge, which is used as a traditional Chinese herbal drug which removes blood stasis, stops pain, and activates blood flow and is heart-relieving [23]. RA shows a great intensity for free radical scavenging and antioxidant activity. The phenylalanine ammonia-lyase (PAL) is the first enzyme for accumulating phenolic acid compounds in* S*.* miltiorrhiza* cell cultures [10], and SA could induce the production of rosmarinic acid [24]. The aim of this work is to reveal the effects of SA and H_2_O_2_ on the accumulation of RA in* S*.* miltiorrhiza* cell cultures. For this purpose, imidazole (IMD, NADPH oxidase inhibitor) is used to inhibit the enzyme activity of NADPH oxidase, and dimethylthiourea (DMTU, H_2_O_2 _scavenger) is employed as scavenger of H_2_O_2_.

## 2. Results

### 2.1. Effects of SA on PAL Activity, H_2_O_2_ Production, and RA Accumulation

**Figure 1:**
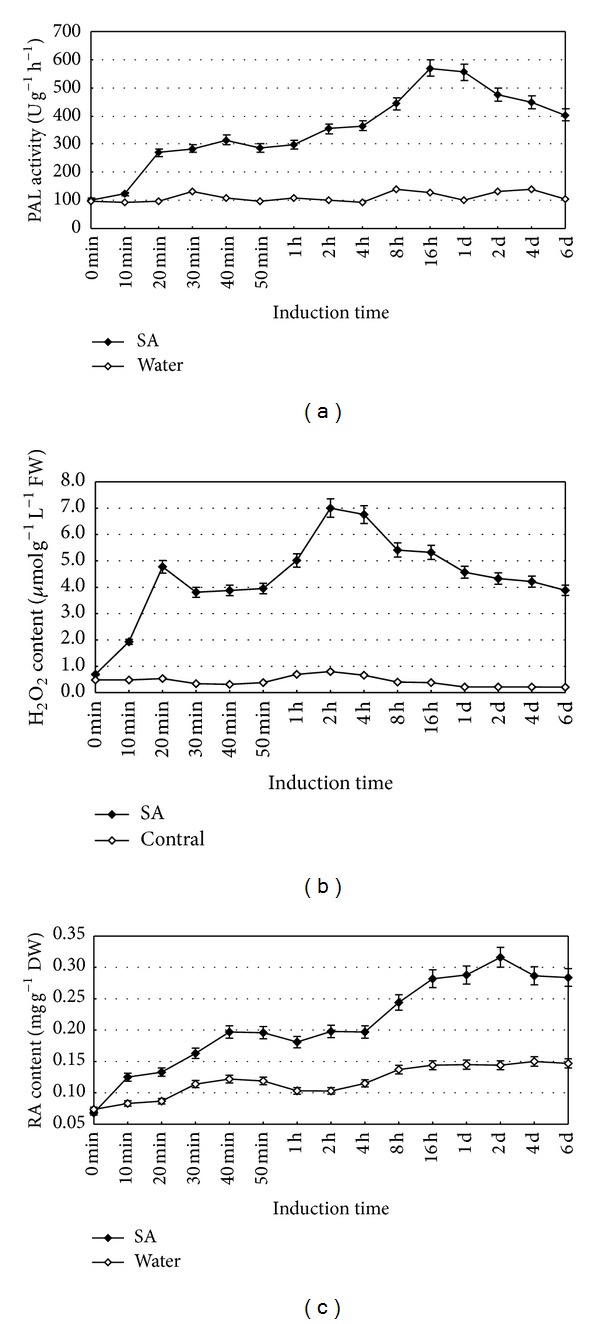
Exogenous SA promotes PAL activity (a), H_2_O_2_ production (b) and RA synthesis (c) in the suspension cultured cells of* S. miltiorrhiza.* 6-day-old suspension cultured cells treated with 22.5 mg/L SA were harvested at the time indicated. The PAL activity and contents of H_2_O_2_ and RA were determined. Values are means of three independent experiments. Bars represented standard errors.

The PAL activity increased from 20 min after treating with 22.5 mg/L SA and reached the peak at 16 h, when the activity was 5.46 folds of that of control, followed by subsequent decrease (Figure 1(a)). The H_2_O_2_ production induced by SA also exhibited continuous increase at the beginning with two peaks, but decreased rapidly after 2-h elicitation. The first peak occurred at 20 min and the second did at 2 h, when 8.51 folds H_2_O_2_ production was found in SA-treated cells (Figure 1(b)). In terms of RA accumulation, continuous increase was found till the 2nd day when RA content reached the highest with 2.15 folds and then it decreased gradually (Figure 1(c)).

### 2.2. Effects of Exogenous H_2_O_2_ on the PAL Activation and RA Production

**Figure 2:**
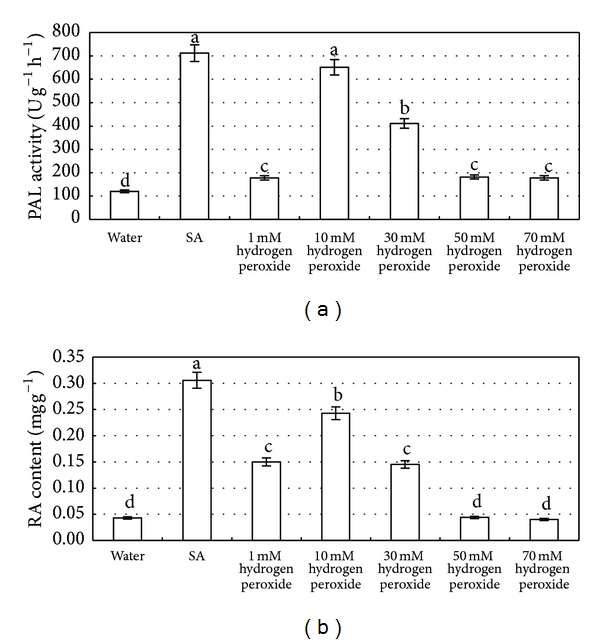
Effects of exogenous hydrogen peroxide with different concentrations on PAL activity (a) and RA accumulation (b) in the* S*.* miltiorrhiza *suspension cultured cells. The salicylic acid concentration is 22.5 mg/L. Values are means of three independent experiments. Bars represented standard errors. Different lowercase letters represent the significant differences between PAL activity (a) and RA content (b) after each treatment at the level of 5%.

The effects of H_2_O_2_ on activation of PAL activity and RA production were investigated, after applying exogenous H_2_O_2 _with concentration ranging from 1 to 70 mM. The activity of PAL and RA content were enhanced with the increase of H_2_O_2_ concentration ranging from 1 to 10 mM, whereas both decreased if the concentration was more than 10 mM (Figure 2). This result suggests that 10 mM is an appropriate concentration to enhance PAL activity and RA accumulation.

**Figure 3:**
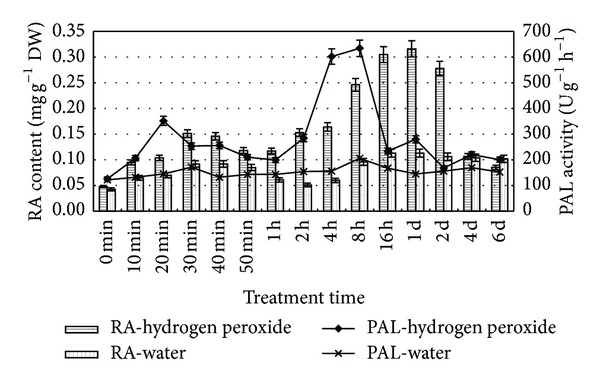
Exogenous hydrogen peroxide promotes PAL activity and RA production in the* S*.* miltiorrhiza *suspension cell cultures. 6-d-old cells treated with 10 mM H_2_O_2_ were harvested at the time as indicated, and RA contents and PAL activities were then determined. Values are means of three independent experiments. Bars represented standard errors.

To further estimate the suitable treatment time of H_2_O_2_, continuous 6-day treatment was performed to confirm the best time point. Both PAL activity and RA accumulation were increased at early stage with time extension and then decreased at the late stage (Figure 3). The activity of PAL reached the highest peak at 8 h, followed by drastic decrease. The RA content reached the peak at 1 d and decreased drastically after 2 d.

### 2.3. Influences of CAT on SA-Induced RA Production

**Figure 4:**
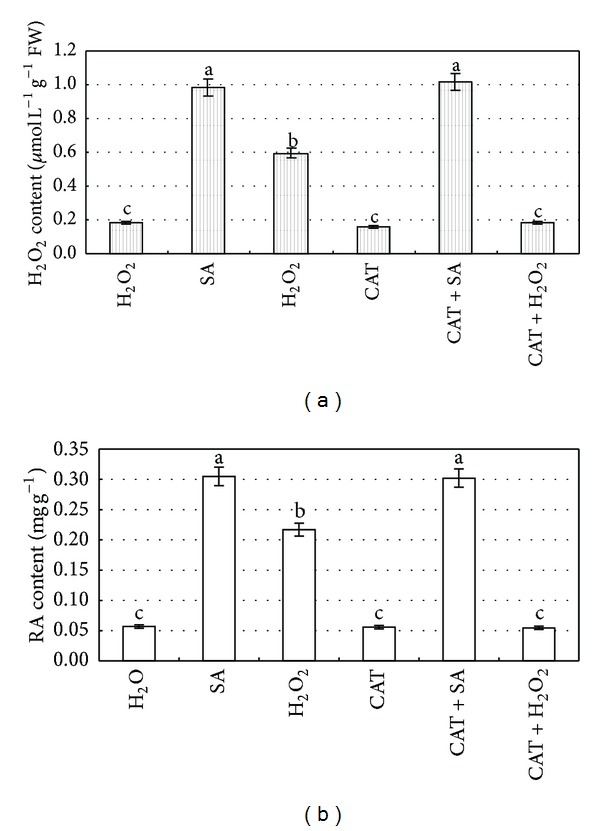
Influences of CAT (a scavenger of H_2_O_2_) on the generation of H_2_O_2_ (a) and the biosynthesis of RA (b) in the suspension cultured cells treated by SA and/or exogenous H_2_O_2_. 6-day-old suspension cultured cells treated with CAT (100 U) 30 min before SA treatment were harvested at 8 h (for H_2_O_2_ analysis) and 2 d (for RA analysis) later, and the H_2_O_2_ and rosmarinic acid contents were then determined. The SA concentration is 22.5 mg/L. The exogenous H_2_O_2_ concentration is 10 mM. Values are means of three independent experiments. Bars represented standard errors. Different lowercase letters represent the significant differences between the concentrations of H_2_O_2_ (a) and RA (b) after each treatment at the level of 5%.

CAT is a scavenger of H_2_O_2_, which can not pass through cell membrane, and thus the exogenous CAT can not scavenge H_2_O_2_ within cells. Both contents of H_2_O_2_ (Figure 4(a)) and RA (Figure 4(b)) were slightly lower than those of control after adding CAT, whereas both contents showed no significant difference between CAT + SA-treated and SA-elicited cells. The application of CAT did not affect the production of endogenous H_2_O_2_ and RA accumulation in cells that were subjected to SA elicitation. However, CAT can scavenge the exogenous H_2_O_2_, thereby eliminating the production of H_2_O_2_ and RA synthesis due to H_2_O_2_ regulation. This indicates that the RA biosynthesis is mediated by intracellular H_2_O_2 _elicited by SA.

### 2.4. Influences of DMTU on SA-Induced RA Production

**Figure 5:**
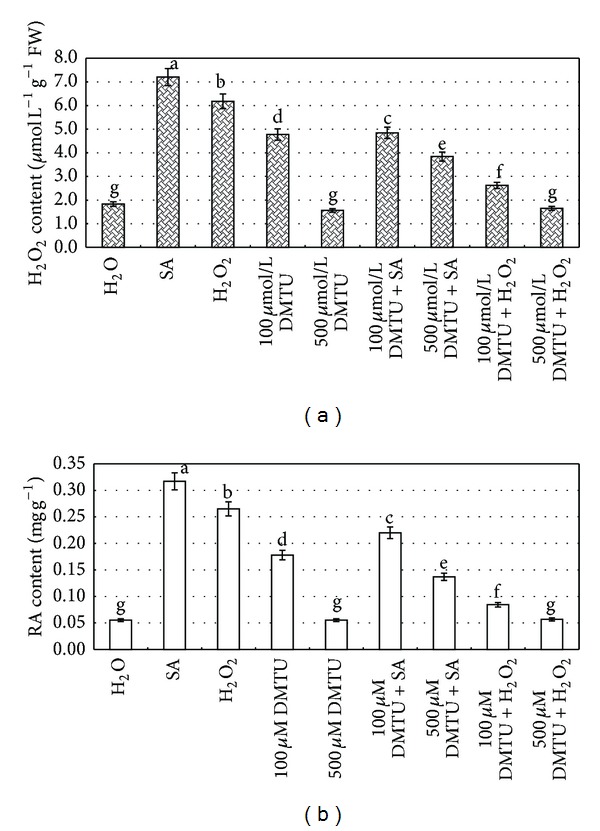
Inhibition of DMTU on exogenous SA in the generation of intracellular H_2_O_2_ (a) and the biosynthesis of RA (b) in suspension cultured cells. 8-day-old suspension cultured cells treated with DMTU 30 min before exogenous H_2_O_2_ and/or SA treatment as indicated were harvested at 8 h (for H_2_O_2_ analysis) and 2 d (for RA analysis) later, and the H_2_O_2_ and Sal B contents were then determined. The Salicylic acid concentration is 22.5 mg/L. The exogenous H_2_O_2_ concentration is 10 mM. Values are means of three independent experiments. Bars represented standard errors. Different lowercase letters represent the significant differences between the contents of H_2_O_2_ (a) and Sal B (b) after each treatment at the level of 5%.

DMTU is a H_2_O_2_ quencher that can effectively remove H_2_O_2_ within cells. The results showed that H_2_O_2_ was released and RA accumulated due to SA elicitation (Figure 1(b)). In order to further explain whether endogenous H_2_O_2_ was involved in the activation of PAL and the synthesis of RA, DMTU was employed to quench endogenous H_2_O_2_, and under such condition, both contents of H_2_O_2 _and RA in cells were measured (Figure 5).

The production of endogenous H_2_O_2_ (Figure 5(a)) and the accumulation of RA (Figure 5(b)) within cells were partially inhibited by 100 *μ*M DMTU, and they were significantly inhibited with the concentration of 500 *μ*M. If the cells were treated by 500 *μ*M DMTU + 22.5 mg/L SA or 500 *μ*M DMTU + 10 mM H_2_O_2_, DMTU would significantly inhibit the production of endogenous H_2_O_2_ and the accumulation of RA. Application of exogenous H_2_O_2_ and/or SA could partially reverse the inhibition of DMTU on RA synthesis. The treatment of 700 *μ*M DMTU also completely inhibited the production of H_2_O_2_ in cells, and the inhibition could not be reversed by application of exogenous SA or H_2_O_2_. These results showed DMTU treatment slightly decreased both contents of RA and H_2_O_2_, when compared with the H_2_O control. Meanwhile, DMTU + SA treatment would significantly block both contents if compared with those SA-elicited cells. These results indicated that H_2_O_2_ produced from SA elicitation is necessary for triggering the biosynthesis of RA.

### 2.5. Influences of IMD on SA-Induced RA Production

NADPH oxidase is one of the key enzymes for the generation of intracellular H_2_O_2_ [25, 26]. IMD is the specific inhibitor of NADPH oxidase on plasma membrane, which can inhibit the production of H_2_O_2_ through inactivating NPDPH oxidase [16, 17, 25]. For instance, IMD scavenged H_2_O_2_ produced from NADPH oxidase elicited by ABA and decreased the synthesis of anthocyanins in rice leaves [21]. In this study, IMD was used to inhibit the activity of NADPH oxidase to examine its influence on the biosynthesis of RA elicited by exogenous H_2_O_2_ and/or SA.

**Figure 6:**
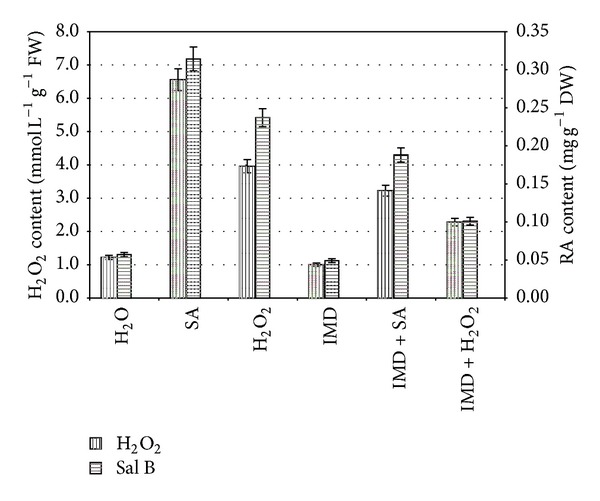
Inhibition of IMD on exogenous H_2_O_2_ and/or SA in the generation of intracellular H_2_O_2_ and the biosynthesis of RA in suspension cultured cells. 8-day-old suspension cultured cells treated with IMD 30 min before exogenous H_2_O_2_ and/or SA treatment as indicated were harvested at 8 h (for H_2_O_2_ analysis) and 2 d (for RA analysis) later, and the H_2_O_2_ and Sal B contents were then determined. The Salicylic acid concentration is 22.5 mg/L. The exogenous H_2_O_2_ concentration is 10 mM. The IMD concentration is 100 *μ*M. Values are means of three independent experiments. Bars represented standard errors.

When IMD (100 *μ*M) was used solely, IMD treatment slightly decreased the contents of RA and H_2_O_2_ when compared with the control, while IMD + SA and IMD + H_2_O_2_ treatments significantly decreased both contents when compared with SA-elicited cells (Figure 6). The results indicated that IMD inhibited the effects of SA elicitation, and the inhibition of IMD to the synthesis of RA in cell cultures depended on H_2_O_2_ generated from NADPH oxidase.

## 3. Discussion

This investigation shows SA can induce the burst of H_2_O_2_, and the elicited H_2_O_2_ enhances the PAL activity and RA accumulation in* S*.* miltiorrhiza* cell cultures. SA signaling pathways in plant defense network have been widely investigated during the past two decades [27, 28], but their role in secondary metabolism is rather limited. Zitta et al. [29] reports that 10 *μ*M SA leads to a significant 4-fold increase in H_2_O_2_ concentrations, which may be attributed to the fact that SA can directly reduce the activity of catalase that decompose of H_2_O_2_ (Figure 4). As in our study, the content of H_2_O_2_ in SA-elicited cells was 8.51-fold higher than that of water control (Figure 1), which is consistent with these reports. Although low level (<10 *μ*M) of H_2_O_2_ is thought to result in the induction of various intracellular signaling pathways [28, 29], application of either exogenous H_2_O_2_ (10 mM) or endogenous H_2_O_2_ elicited by SA (22 mg/L) showed its capacity to enhance both PAL activity and RA accumulation in our experiments. PAL is the first key enzyme in the phenylpropanoid pathway for the biosynthesis of RA [30], which has been reported to be enhanced by SA in the colonization of arbusclular mycorrhizal fungus [28] and in the interaction between tomato plants and* Fusarium oxysporum* f. sp.* lycopersici* [31]. H_2_O_2_ comes to light as a second messenger involved in SA-elicited pathway. In this paper, both PAL activity and RA accumulation were significantly increased when exogenous 10 mM H_2_O_2_ was applied (Figure 2). On the other hand, H_2_O_2_ could be elicited by SA even though it was treated by catalase or DMTU (Figures 4 and 5), and the elicited H_2_O_2_ also could significantly promote RA accumulation. Our results are consistent with those reports in the fact that H_2_O_2_ is involved in many elicitor-induced processes to produce secondary metabolites, for instance, the biosynthesis of taxol [19], catharanthine [20], anthocyanidins [21], and anthraquinone [22].

Increasing evidence proved that plant responses to elicitors, including enzymes activation and the production of secondary metabolites, are not only regulated by signaling pathway, but also a cross-talk process. The mechanism about cross-talk among SA, H_2_O_2_, enzyme activity, and synthesis of secondary metabolites has gained some attention recently, but related studies are rare [32]. According to the latest progress in our lab, both nitric oxide (NO) and increase of intracellular Ca^2+^ are also involved in this cross-talk during SA induction.

Taken together, H_2_O_2_ production can be elicited by SA and the elicited H_2_O_2_ can promote PAL activity and RA accumulation, in which H_2_O_2_ plays an important role as a second messenger in signal transduction when SA is applied.

## 4. Experimental Section

### 4.1. Cell Culture

The detailed protocols of* S*.* miltiorrhiza* cell culture were provided by Dong et al. [10]. The seeds were collected from Shangluo, Shaanxi Province, and then soaked in water for 2–4 h. The wax coat was removed by gauze, followed by washing with water. The seeds were soaked in 70% ethanol for 30 s. After washing by sterile water for 3 times, the seeds were sterilized by 0.1% HgCl_2_ for 10–15 min. They were sown in the autoclaved MS solid media after washing with sterile water for three times, supplemented with 5.5 g/L agar (Beijing Kangbeisi Sci & Tech Company) and 30 g/L sucrose (Guangzhou Jinhuada Chemical Company) to induce the germ free seedlings. The leaves of germ free seedlings were cut into 0.5 cm × 0.5 cm pieces and inoculated on the autoclaved MS solid medium supplemented with 1.0 mg/L NAA (Tianjin Bodi Chemical Company), 1.0 mg/L 6-BA (Beijing Kangbeisi Sci & Tech Company), 1.0 mg/L 2,4-D (Beijing Kangbeisi Sci & Tech Company), 5.5 g/L agar, and 30 g/L sucrose. Calli were induced and cultured at 25 ± 2°C with light intensity of 2000–3000 Lx for 12–16 h per day. After 15-day culture, the calli would be subcultured till two months when their morphological characteristics and growth rates were stable. The stable calli were collected and inoculated in MS liquid media (containing 30 g/L sucrose) with the ratio of callus to culture medium 1 : 15 (W/V). The suspension cells were cultured in the dark at 25°C with shaking speed of 125 rpm.

### 4.2. Elicitation and Chemical Treatments

Stock solutions of SA, H_2_O_2_, DMTU (H_2_O_2_ scavenger, Sigma, USA), CAT (Sigma, USA), and IMD (NADPH scavenger, Sigma, USA) were prepared in distilled water and then sterilized after filtration through 0.22 *μ*m membrane. The resultant solutions with different concentrations were added in the media in accordance with the experimental design. In the control group, only distilled water with the same volume was added to substitute for the chemicals. DMTU, CAT, and IMD were added separately to the cell culture 30 min before SA treatment. All experiments were performed with three replications.

### 4.3. PAL Activity Assay

PAL activity was measured according to the method of Solecka and Kacperska [33] with some modifications. Fresh cultured calli were placed on filter paper in a Bucher funnel, filtered in vacuum, washed by distilled water, and filtered in vacuum again to remove the facial water. Two grams of calli were homogenized with 5 mL extraction buffer in a mortar. The homogenate was filtered through four-layer cheesecloth and centrifuged at 10000 rpm for 15 min at 4°C to get supernatant that would be used as a crude enzyme. The reaction mixture (3 mL) included 0.5 mL crude enzyme, 16 mM L-phenylalanine, 50 mM Tris-HCl buffer (pH 8.9), and 3.6 mM NaCl. The mixture was incubated at 37°C for 60 min and the reaction was stopped by 500 *μ*L 6 M HCl. The resultant mixture was then centrifuged (12,000 ×g, 10 min). The absorbance was measured at 290 nm before and after incubation. The enzyme activity (*U*) was calculated by the following equation:

(1) $$\begin{array}{c} \text{PAL}\left(U\cdot g^{-1}\text{FW}\cdot h^{-1}\right)=\frac{A_{290}\times v_{T}\times v}{0.01\times V_{s}\times \text{FW}\times t}, \end{array}$$

where *V*_*t*_ represents the total volume of enzyme solution (mL); FW is the fresh weight of calli; *V*_*s*_ is the volume of the enzyme solution sampled (mL); *ν* is the total volume of the reaction solution (mL); and *t* is the reaction time (*h*).

### 4.4. Determination of Hydrogen Peroxide

Hydrogen peroxide was determined by the method of Li et al. [34]. The calli (0.2-0.3 g) were homogenized with 5 mL acetone precooled under 4°C and then ground to homogenate on the ice. The mixture was centrifuged at 12,000 rpm for 10 min at 4°C. The supernatant (2 mL) was mixed quickly with 0.5 mL 5% titanium sulfate, and ammonia solution (2 mL) was added and homogenized. The resultant mixtures were then centrifuged again; the supernatant was discarded. The precipitate (the titanium-hydro peroxide complex) was dissolved in 5 mL 2 M sulfuric acid, and the supernatant absorbance was measured at 415 nm, and the content of hydrogen peroxide was calculated.

### 4.5. RA Extraction and HPLC Analysis

The* S*.* miltiorrhiza* cells were collected from cell cultures by centrifugation at 1200 rpm, and then dried at 47.5°C in an oven till constant weight. The dried cells (0.05 g) were put into a test tube with a stopper, and the extraction was conducted with aqueous methanol (70 : 30, methanol : water) for 45 min in an ultrasonic bath. The extract was filtered through a 0.22 *μ*m membrane and the filtrate was obtained for detection.

The content of RA was quantified by HPLC (Shimadzu, model SCL-10AVPTM equipped with UV/Vis absorbance detector, 150 mm × 4.6 mm shim-pack column, and LC-ATVP pump). The mobile phase was acetonitrile : water : phosphate (25 : 75 : 0.1, v/v/v). The flow rate was 1 mL/min and the injection amount was 10 *μ*L. The detection wavelength was 285 nm and the column temperature was 25°C.

## 5. Conclusions

SA is an effective elicitor inducing RA accumulation in* S. miltiorrhiza* cell cultures, which can act independently or synergistically with H_2_O_2_. The intracellular H_2_O_2_ acts as a signal molecule to participate in RA accumulation in response to SA elicitation.
